# Severe Anemia (1.4 g/dL) Due to Uterine Fibroid Bleeding With Reversible Myocardial Dysfunction: A Case Report

**DOI:** 10.7759/cureus.100528

**Published:** 2025-12-31

**Authors:** Houmed Houssein Houmed, Mohamed Benani, Manal Arfaoui, Elachab Nabil, Rajae Tachinante

**Affiliations:** 1 Anesthesiology and Intensive Care, Mohammed V Military Teaching Hospital, Mohammed V University, Rabat, MAR; 2 Anesthesiology and Critical Care, Maternity Souissi Hospital, Ibn Sina University Hospital, Rabat, MAR

**Keywords:** abnormal uterine bleeding, reversible myocardial dysfunction, severe anemia, stress induced cardiomyopathy, uterine leiomyoma

## Abstract

Abnormal uterine bleeding (AUB), particularly when associated with uterine leiomyomas, is a frequent cause of iron deficiency anemia among women of reproductive age. While commonly manageable, in rare instances, AUB can result in critically low hemoglobin levels, with potentially life-threatening consequences. We report a rare case of a patient presenting with a hemoglobin concentration of 1.4 g/dL secondary to fibroid-induced hemorrhage, complicated by acute but reversible myocardial dysfunction.

We describe the case of a 41-year-old North African woman with a known history of uterine fibroids and endometriosis, who presented to the emergency department with profuse AUB and altered consciousness. On admission, she was hemodynamically unstable with severe anemia (hemoglobin 1.4 g/dL, hematocrit 5.1%), coagulopathy, and metabolic acidosis. Pelvic ultrasound revealed two fibroids, one submucosal and one pedunculated. The patient received aggressive resuscitation, including transfusion of 6 units of packed red blood cells and 5 units of fresh frozen plasma intraoperatively with norepinephrine support (0.3-0.8 µg/kg/min), followed by total hysterectomy. Postoperatively, she developed respiratory distress diagnosed as cardiogenic pulmonary edema with acute left ventricular systolic dysfunction (ejection fraction 28%), definitively distinguishing it from Acute Respiratory Distress Syndrome (ARDS) and Transfusion-Related Acute Lung Injury (TRALI) based on echocardiographic findings, clinical presentation, and rapid response to diuretic therapy. Medical management led to gradual improvement, and one month later, her ejection fraction normalized to 50%.

This case clearly illustrates the remarkable ability of the human body to adapt under extreme hematological stress caused by uterine fibroid-related hemorrhage. Serial echocardiographic documentation demonstrates complete reversibility of anemia-induced cardiomyopathy. It also demonstrates how timely diagnosis and coordinated medical intervention can effectively prevent severe complications, including permanent myocardial damage. While 23 published cases with hemoglobin <2 g/dL exist in medical literature, only three cases at the 1.4-1.6 g/dL level have been documented, underscoring this case's extreme rarity and educational value.

## Introduction

Uterine fibroids, also known as myomas or leiomyomas, are benign uterine tumors originating from smooth muscle cells. They represent a common gynecological condition among women of reproductive age, with an estimated prevalence between 8% and 18%. The most frequent clinical manifestation associated with fibroids is chronic bleeding, which can result in iron-deficiency anemia as a major consequence.

Severe anemia represents a critical medical emergency with mortality increasing exponentially below hemoglobin levels of 5 g/dL [1,2]. Esteves et al. identified 23 published case reports documenting survival with hemoglobin <2 g/dL [3], with only three cases at the 1.4-1.6 g/dL level [4,5,6]. Mortality approaches 60%-80% at hemoglobin levels <3 g/dL, with Carson et al. reporting 100% mortality for hemoglobin 1-2 g/dL in their surgical cohort who refused transfusion [2,3]. While uterine fibroid bleeding is a common cause of chronic anemia, progression to life-threatening levels represents a complex interplay of delayed recognition, limited healthcare access, and physiological adaptation [7].

The medical literature reports rare instances of severe anemia associated with uterine fibroids, with hemoglobin (Hb) levels below 2 g/dL, occasionally complicated by reversible cardiogenic shock. However, none of these cases provided complete serial echocardiographic documentation demonstrating cardiac recovery. This case provides comprehensive quantitative left ventricular ejection fraction (LVEF) measurements at Days 1 and 30, documenting complete reversibility of anemia-induced cardiomyopathy - a level of documentation rarely achieved in published literature.

This case demonstrates remarkable human physiological adaptation at approximately 10% of normal oxygen-carrying capacity and provides a systematic approach to differential diagnosis of postoperative respiratory complications, clearly distinguishing cardiogenic pulmonary edema from Acute Respiratory Distress Syndrome (ARDS) and Transfusion-Related Acute Lung Injury (TRALI). Furthermore, it offers valuable evidence supporting goal-directed transfusion strategies in extreme anemia when hemodynamic stability can be maintained with appropriate vasopressor support. This article presents this exceptional case with comprehensive documentation of management and complete cardiac recovery.

## Case presentation

A 41-year-old unmarried woman with a four-year history of a polymyomatous uterus and endometriosis presented with AUB. She had previously been treated for iron-deficiency anemia with oral iron therapy, which was discontinued, and she did not receive adequate follow-up, having been lost to follow-up for the past three years.

She initially presented to a private clinic with profuse AUB accompanied by impaired consciousness and was subsequently transferred to the emergency unit of Soussi Maternity Hospital in Rabat, Morocco.

Upon admission, she was found to be hemodynamically unstable and confused, with a Glasgow Coma Scale score of 10/15 (E2V3M5). Initial automated oscillometric blood pressure (BP) measurements were unobtainable due to severe shock. Clinical estimation suggested profound hypotension (based on a weak peripheral pulse and altered consciousness). The first measurable blood pressure was 55/30 mmHg after an initial fluid bolus of 500 mL crystalloid. She was tachypneic (respiratory rate 32 breaths/min), tachycardic (heart rate 135 bpm), markedly pale, and had decolorized conjunctivae.

Ultrasound and laboratory investigations

**Figure 1:**
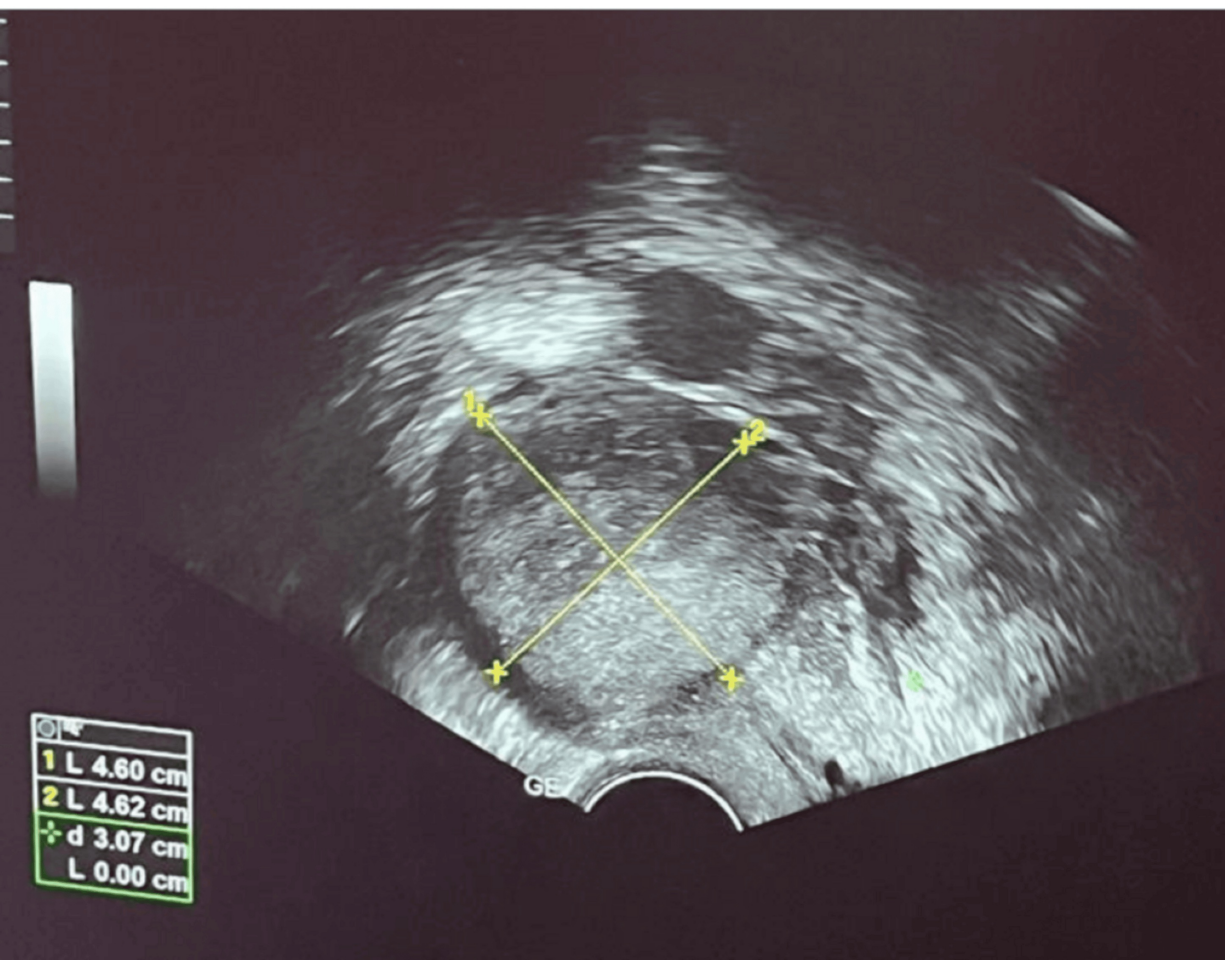
Pelvic ultrasound demonstrating FIGO type 2-5 submucosal fibroid measuring 4.6 × 4.6 cm in the anterior fundal region. FIGO, International Federation of Gynecology and Obstetrics

**Figure 2:**
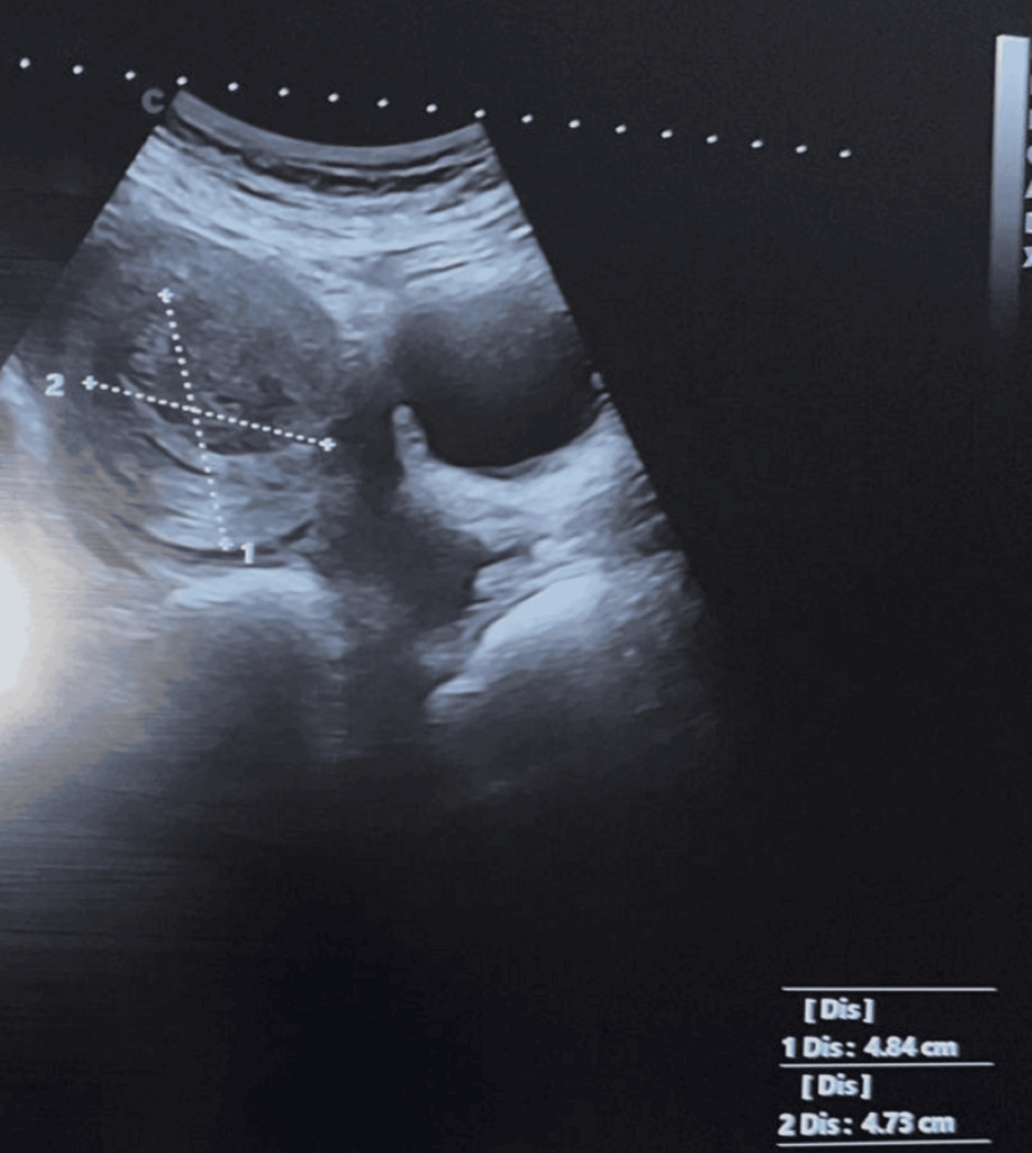
Pelvic ultrasound showing FIGO type 6 pedunculated fibroid measuring 4.8 × 4.7 cm at the fundus. FIGO, International Federation of Gynecology and Obstetrics

Pelvic ultrasound revealed an enlarged uterus containing two fibroids: one International Federation of Gynecology and Obstetrics (FIGO) type 2-5 fibroid measuring 4.6 × 4.6 cm in the anterior fundal region, and another FIGO type 6 pedunculated fibroid measuring 4.8 × 4.7 cm at the fundus. Abdominal and pelvic ultrasound also showed a small volume of intra-abdominal fluid in the perivascular and pelvic regions (Figures 1-2).

Admission laboratory findings revealed hemoglobin of 1.4 g/dL (normal 12-16 g/dL), hematocrit of 5.1% (normal 36%-48%), mean corpuscular volume of 118 fL (macrocytic), and a platelet count of 98,000/µL (mildly decreased). Blood samples for laboratory analysis were drawn 3 minutes after emergency department (ED) arrival, with only 500 mL of crystalloid administered during prehospital transport, definitively excluding dilutional anemia. Coagulation tests showed an international normalized ratio (INR) of 3.43 (normal 0.8-1.2) and an activated partial thromboplastin time of 58 seconds. Rapid fibrinogen assay was unavailable at admission due to an emergency. Coagulation was corrected empirically with fresh frozen plasma (FFP, 5 units). The first measurement during surgery showed a fibrinogen level of 1.8 g/L (normal 2-4 g/L), which was considered adequate without the need for additional fibrinogen supplementation.

Arterial blood gas analysis confirmed severe metabolic acidosis: pH 6.88 (normal 7.35-7.45), PaCO₂ 39 mmHg, PaO₂ 69.4 mmHg, HCO₃⁻ 7.3 mmol/L, base excess -22.8 mmol/L, lactate 6.32 mmol/L (normal 0.5-2.2 mmol/L). The profound metabolic acidosis and marked hyperlactatemia were secondary to severe global tissue ischemia from hemorrhagic shock [8]. Renal function showed urea 0.48 g/L and creatinine 9.8 mg/L (mildly elevated).

The elevated INR resulted from consumptive coagulopathy secondary to multiple factors: hepatic hypoperfusion during hemorrhagic shock reducing synthesis of vitamin K-dependent coagulation factors (II, VII, IX, X); consumption of clotting factors in ongoing bleeding; profound metabolic acidosis (pH 6.88) causing >50% reduction in coagulation enzyme activity - at pH <7.0, coagulation cascade enzymes demonstrate markedly impaired function; and hypothermia (temperature 35.8 °C on admission) further impairing enzymatic coagulation reactions. The rapid normalization of INR (3.43 → 1.65 at 6 hours → 1.32 on Day 1 → 1.12 on Day 3 → 0.98 on Day 7) following correction of shock, acidosis, and hypothermia confirms the diagnosis of reversible shock-related coagulopathy rather than a primary coagulation disorder [8].

Emergency management and surgery

After initial hemodynamic stabilization with norepinephrine support, rapid sequence induction was performed using ketamine 50 mg intravenous (IV) (0.65 mg/kg) combined with fentanyl 70 µg IV (1 µg/kg). Ketamine was specifically selected for its favorable hemodynamic profile, maintaining sympathetic tone with minimal myocardial depression, both critical considerations in severe hemorrhagic shock. Neuromuscular blockade was achieved with rocuronium 70 mg IV (1.2 mg/kg). Anesthetic maintenance consisted of sevoflurane 0.8%-1% minimum alveolar concentration (MAC) with intermittent ketamine boluses and fentanyl boluses (50 µg prn) for balanced anesthesia.

Comprehensive invasive monitoring was established, including a right radial arterial line for continuous BP monitoring and a right internal jugular central venous line for central venous pressure (CVP) monitoring and central venous oxygen saturation (ScvO₂) assessment. Hourly laboratory assessments (arterial blood gases, lactate, hemoglobin, coagulation panel) guided transfusion and hemodynamic management throughout the perioperative period.

Intraoperative vasopressor support was maintained with norepinephrine 0.3-0.8 µg/kg/min, targeting mean arterial pressure >65 mmHg and titrated based on continuous arterial pressure monitoring, CVP, and ScvO₂. Postoperatively, norepinephrine weaning was initiated immediately, with complete discontinuation within the first few hours as hemodynamics stabilized and hemoglobin levels improved.

The transfusion protocol consisted of 6 units of packed red blood cells (PRBC) and 5 units of FFP (ratio 1:1.2) administered intraoperatively, with all products warmed to 37 °C using a rapid infuser. Two additional units of PRBC were transfused on postoperative Day 1, totaling 8 units of PRBC and 5 units of FFP. This goal-directed transfusion strategy was guided by hemodynamic parameters and serial hemoglobin monitoring, successfully avoiding massive transfusion complications while ensuring adequate tissue oxygenation.

Hemostatic management included tranexamic acid 1 g IV bolus at induction, followed by a continuous infusion of 1 g every 8 hours for 24 hours. Antibiotic prophylaxis was provided with cefazolin 2 g at induction, followed by 1 g every 4 hours intraoperatively.

A total hysterectomy was performed via midline laparotomy, with a duration of 5 hours and an estimated blood loss of 400 mL. The patient remained hemodynamically stable throughout the surgical procedure with appropriate vasopressor support and judicious transfusion strategy.

Postoperative course

Early postoperative monitoring at 10 hours demonstrated significant improvement across all parameters. Hemoglobin increased to 6.8 g/dL with hematocrit 20.4%, while coagulation showed rapid correction with INR declining to 2.02 and prothrombin time improving to 37%. Metabolic acidosis substantially improved with pH 7.2, lactate 3.2 mmol/L, and PaO₂ 201 mmHg on supplemental oxygen. This early improvement validated the effectiveness of immediate resuscitation and guided subsequent management. Serial biological parameters evolution is detailed in Table 1.

**Table 1:** Serial biological parameters and transthoracic echocardiography evolution. Progressive normalization of all parameters was documented from admission through Day 30, with complete cardiac recovery demonstrated by LVEF improvement from 28% to 50% at one-month follow-up. *Fibrinogen measured intraoperatively, not on admission. - = Not measured at this time point. N/A = not available. aPTT, activated partial thromboplastin time; INR, international normalized ratio; PT, prothrombin time; LVEF, left ventricular ejection fraction Units of measurement: fL, femtoliters; g/dL, grams per deciliter; g/L, grams per liter; mg/L, milligrams per liter; mmHg, millimeters of mercury; mmol/L, millimoles per liter; µg, micrograms; µL, microliter

Parameter	Admission	H10 post-op	Day 1	Day 3	Day 30	Reference range
Hematology						
Hemoglobin (g/dL)	1.4	6.8	8.1	9.8	-	12.0-16.0
Hematocrit (%)	5.1	20.4	24.4	29.4	-	36.0-48.0
Platelets (/µL)	98	149	118	120	-	150,000-400,000
Coagulation						
PT (%)	-	37	62	65	-	70-100
INR	3.43	2.02	1.36	1.12	0.98 (Day 7)	0.8-1.2
aPTT ratio	-	1.01	1.0	1.1	-	0.8-1.2
Fibrinogen (g/L)	-	1.8*	-	2.3	3.1	2.0-4.0
Renal function						
Urea (g/L)	0.48	0.8	0.43	-	-	0.15-0.45
Creatinine (mg/L)	9.8	9.7	8.0	8.9	-	6.0-12.0
Arterial blood gas						
pH	6.88	7.2	7.38	7.40	-	7.35-7.45
PaO₂ (mmHg)	69.4	201	180	-	-	80-100
PaCO₂ (mmHg)	39	42	37.2	-	-	35-45
Lactate (mmol/L)	6.32	3.2	1.93	0.85	-	0.5-2.2
Transthoracic echocardiography						
LVEF (%)	N/A	-	28	-	50	≥55

Approximately 23 hours postoperatively (one hour after administration of the 2 additional PRBC units on Day 1), the patient developed acute respiratory distress: dyspnea, tachypnea (respiratory rate 34 breaths/min), oxygen saturation 82% on room air requiring oxygen supplementation (FiO₂ 50% via face mask), and bilateral crackles on auscultation. Hemodynamically, she remained stable with BP 118/76 mmHg and heart rate 98 bpm (no vasopressor requirement).

Diagnostic workup

Transthoracic echocardiography (Day 1) revealed severe left ventricular (LV) systolic dysfunction with an ejection fraction of 28% and a restrictive mitral inflow pattern (E/A ratio >2), confirming cardiogenic pulmonary edema. No valvular abnormalities or pericardial effusion were identified. Laboratory investigations showed hemoglobin 8.1 g/dL, lactate 1.93 mmol/L, pH 7.38, INR 1.36, and creatinine 8.0 mg/L (Video 1).

**Video 1:** Transthoracic echocardiogram (apical 4-chamber view) demonstrating severe global hypokinesia and marked left ventricular systolic dysfunction.

Comprehensive differential diagnosis

The diagnosis of cardiogenic pulmonary edema secondary to anemia-induced reversible cardiomyopathy was established based on systematic exclusion of alternative diagnoses. ARDS was excluded based on multiple criteria: the timing of respiratory distress onset, which occurred less than 24 hours after the inciting event, whereas ARDS typically develops 24-48 hours post-insult; the presence of severe left ventricular (LV) dysfunction documented by echocardiography, contrasting with the normal or hyperdynamic LV function characteristic of ARDS; the rapid response to diuretic therapy, which would not occur in ARDS due to underlying alveolar injury; and the absence of systemic inflammatory response syndrome criteria. TRALI was similarly excluded based on compelling evidence: the timing of respiratory distress at 23 hours after the last transfusion far exceeded the required 6-hour window for TRALI diagnosis, which typically manifests within 1-2 hours; the absence of fever, whereas TRALI characteristically presents with temperatures exceeding 38.5°C; documentation of severe LV dysfunction, which does not occur in TRALI pathophysiology; maintenance of hemodynamic stability, contrasting with the acute hypotension typical of TRALI; and progressive improvement with heart failure treatment rather than the spontaneous resolution within 48-96 hours expected in TRALI. These systematic exclusions confirmed that the respiratory distress represented cardiogenic pulmonary edema secondary to reversible anemia-induced cardiomyopathy.

Cardiac management and outcome

Treatment consisted of furosemide 40 mg IV bolus followed by a continuous infusion of 5 mg/hour, fluid restriction to <1,000 mL/day, oxygen at 3-5 L/min via nasal cannula, bisoprolol 2.5 mg daily, ramipril 2.5 mg daily (titrated to 5 mg), and spironolactone 25 mg daily. No inotropic support was required; management focused on diuretic therapy, afterload reduction, and oxygen supplementation.

Clinical evolution demonstrated rapid improvement. Oxygen therapy was weaned on Day 4. Complete resolution of pulmonary edema by Day 7. Serial echocardiography documented progressive improvement: LVEF of 28% on Day 1 recovered to 50% at one-month follow-up, confirming complete reversibility of anemia-induced cardiomyopathy. The patient was discharged on Day 10 in stable condition (hemoglobin 10.2 g/dL, INR 0.98). At one-month follow-up, complete cardiac recovery was confirmed (LVEF 50%), validating the diagnosis of reversible anemia-induced cardiomyopathy (Video 2).

**Video 2:** Follow-up transthoracic echocardiogram (apical view) at one month showed normalization of left ventricular systolic function, with an estimated ejection fraction of 50%-52%.

## Discussion

AUB can have various etiologies, often classified under the FIGO PALM-COEIN system. In our case, pelvic ultrasound identified uterine fibroids as the source of life-threatening anemia (hemoglobin 1.4 g/dL) [7]. While 23 published case reports document survival with hemoglobin <2 g/dL per the comprehensive 2021 review by Esteves et al., only three cases have reported levels specifically within the 1.4-1.9 g/dL range: Qaadri et al. (1.6 g/dL), Chai et al. (1.4 g/dL), and Kawano et al. (1.9 g/dL) [3,4,5,6]. Our case stands alongside that of Chai et al. for the lowest hemoglobin concentration ever documented at 1.4 g/dL [5]. Mortality at hemoglobin <3 g/dL is exceptionally high, with rates exceeding 50%-80%. Carson et al. demonstrated that operative mortality increases exponentially with decreasing hemoglobin levels, approaching 100% in surgical patients who refuse transfusion when levels fall below 2 g/dL [9]. Critically, none of these previously reported cases provided comprehensive serial echocardiographic documentation of cardiac recovery. Our case provides a complete quantitative assessment at Days 1 and 30, demonstrating full reversibility (LVEF 28%-50%) - a level of documentation rarely achieved in published literature.

Management options for symptomatic fibroids include uterine artery embolization for fertility preservation or total hysterectomy for definitive resolution. Given the patient's age (41 years), absence of fertility desires, profound hemodynamic instability (Class IV hemorrhagic shock), and critical need for immediate definitive hemostasis, total hysterectomy was the appropriate intervention [7].

Although the total volume of PRBC units transfused (8 units over 24 hours) remains below the historical definition of massive transfusion (traditionally ≥10 units/24 hours), the management aligns with modern, dynamic definitions of critical hemorrhage [10]. Specifically, with 6 PRBC units administered during the 5-hour intraoperative period, the patient met the Critical Administration Threshold (CAT) - defined as the transfusion of ≥3 units within one hour - as well as the *5 units in 4 hours* criterion [10].

Furthermore, when considering the global Resuscitation Intensity (RI), the total blood products reached 13 units (8 PRBC and 5 FFP). This proactive approach, prioritizing an early PRBC:FFP ratio of 1:1.2, successfully restored oxygen transport while preventing aggravation of shock-related coagulopathy [8,11].

This targeted strategy, tailored to the severity of hemorrhagic shock, contrasts with the strictly restrictive approach (2 units) described by Esteves et al. in their patient with chronic vitamin B12 deficiency anemia, highlighting the importance of individualizing transfusion based on acute clinical presentation - hemorrhagic shock versus chronic adaptation [3].

**Figure 3:**
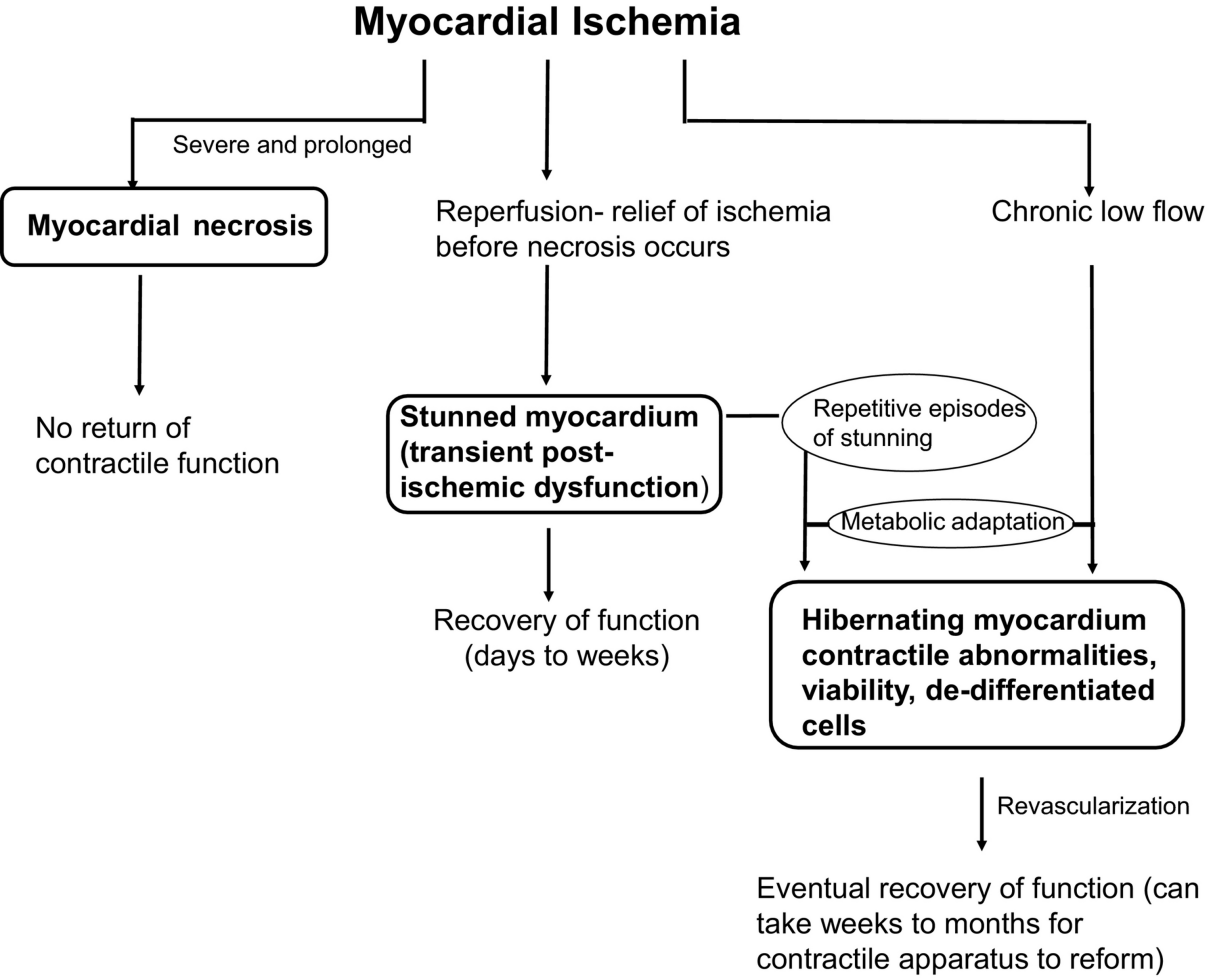
Pathophysiological outcomes of myocardial ischemia: from necrosis to reversible dysfunction including stunning and hibernation. Open access journal under a CC BY-NC-ND 4.0 license contributed by Kloner RA. Stunned and Hibernating Myocardium: Where Are We Nearly 4 Decades Later? [14].

The transient myocardial dysfunction observed represents anemia-induced reversible cardiomyopathy or myocardial *stunning* - a prolonged, reversible post-ischemic ventricular dysfunction occurring without irreversible necrosis [12,13,14]. The pathophysiology involves a profound oxygen supply-demand mismatch, causing transient contractile dysfunction while preserving myocyte viability [15]. Complete reversibility (LVEF 28%-50%) at one month confirms the absence of permanent ischemic injury. The schematic in Figure 3 documents three possible outcomes of myocardial ischemia [14]. On the left is the situation of severe and prolonged myocardial ischemia. The myocardial cells die resulting in a myocardial infarction, are replaced by scar tissue, and do not recover contractile function. In the middle is the scenario in which the duration and severity of myocardial ischemia are not long enough or severe enough to kill cells. When the ischemia is relieved by reperfusion, the myocardium is viable but stunned, exhibiting transient post-ischemic contractile and biochemical dysfunction. Recovery of the stunned myocardium eventually occurs but may take days to weeks. The third scenario of ischemia is shown on the right. Chronic low blood flow results in metabolic adaptations allowing the cardiomyocytes to survive, but these cells do not contract at rest and exhibit typical morphology of dedifferentiation. Once revascularized, these hibernating myocardial cells eventually recover function, but this may require weeks to months as the contractile apparatus replenishes. In addition, another theory of hibernating myocardium is shown in which repetitive episodes of ischemic, stunned myocardium occur when coronary artery reserve cannot meet an increase in myocardial oxygen demand. These repetitive episodes of stunning lead to a chronic reduction in contractile function and metabolic adaptation to allow for cell survival [14,15].

The systematic approach to differential diagnosis was crucial for optimal management. Distinguishing cardiogenic pulmonary edema from ARDS and TRALI has profound therapeutic implications: cardiogenic edema responds rapidly to diuretics and afterload reduction, whereas ARDS and TRALI require supportive care [16]. The diagnosis of cardiogenic pulmonary edema was confirmed by echocardiography, demonstrating severe LV dysfunction (LVEF 28%) with a restrictive mitral inflow pattern, rapid clinical response to diuretics (oxygen weaned by Day 4), and complete recovery of LVEF to 50% at one month [15,16]. ARDS and TRALI were definitively excluded based on timing, echocardiographic findings, and clinical evolution, as detailed in the Case Presentation section. The lack of comorbidities and spontaneous complete recovery strongly support reversible anemia-induced cardiomyopathy rather than underlying structural heart disease unmasked by stress.

## Conclusions

This case demonstrates that survival from extreme anemia (hemoglobin 1.4 g/dL) is achievable through prompt recognition, aggressive resuscitation, judicious transfusion strategy tailored to clinical presentation, and definitive surgical hemostasis. The systematic approach to differential diagnosis, distinguishing cardiogenic pulmonary edema from ARDS and TRALI based on echocardiographic findings and therapeutic response, was crucial for optimal management.

Complete documentation of cardiac recovery (LVEF 28% → 50%) with serial echocardiography provides valuable evidence that anemia-induced cardiomyopathy is completely reversible. This supports individualized transfusion strategies even in extreme anemia when hemodynamic stability can be maintained with vasopressor support.

Early therapeutic intervention, comprehensive hemodynamic monitoring, and serial echocardiographic assessment are essential to optimize outcomes. The extreme rarity of this case, one of only three documented cases at the 1.4-1.6 g/dL level with complete serial echocardiographic documentation, makes it a valuable educational resource for clinicians.

Further studies are needed to understand the prevalence and mechanisms of reversible myocardial dysfunction in massive hemorrhagic events and establish evidence-based guidelines for transfusion thresholds and cardiac management in extreme anemia.
